# Self reported experience of sexual abuse among children in their homes in Ilorin Nigeria

**DOI:** 10.1192/j.eurpsy.2021.1891

**Published:** 2021-08-13

**Authors:** A. Oladosu, O. Abiodun, M. Tunde-Ayinmode

**Affiliations:** 1 Johnson Community Hospital, Lincolnshire Partnership Foundation Trust, Lincolnshire, United Kingdom; 2 Behavioural Sciences, University of Ilorin Teaching Hospital, Ilorin, Nigeria

**Keywords:** sexual abuse, Child, home

## Abstract

**Introduction:**

The sexual abuse of children is well documented in literature. Data on it from Nigeria is rather sparse. The current study examines the prevalence and pattern of sexual abuse with a view to increasing our understanding of it.

**Objectives:**

To determine the prevalence and pattern of sexual abuse of children at home in Ilorin Nigeria.

**Methods:**

A cross sectional survey of secondary school students aged 11-18 years in Ilorin Nigeria using multistage random sampling technique with proportional allocation was done. Respondents completed the ICAST-CH questionnaire which covers child abuse in its several forms including sexual abuse. Prevalence of sexual abuse was computed.

**Results:**

Over a third (586) of participants experienced some form of sexual abuse in the last year. Table 1: prevalence and pattern of sexual abuse at home

**Table:** 

Sexual Abuse* (n=586)	Frequency	Percentage
Talked to you in a sexual way	420	71.7
Touched private parts	333	56.8
Showed pornography	149	25.4
Made you look at private parts	136	23.2
Tried to have sex with you (unwilling)	61	10.4
Made a sex video of you	-	-

**Conclusions:**

Sexual abuse of children occurs commonly in Ilorin Nigeria. There is a need for further research towards understnding it determinants towards strengthening systems of safeguarding children against it.

**Disclosure:**

No significant relationships.

